# Wisconsin Society of Veterinary Graduates

**Published:** 1895-04

**Authors:** W. G. Clark

**Affiliations:** Secretary


Secretary.

### WISCONSIN SOCIETY OF VETERINARY GRADUATES.

The fifth annual meeting of the Society was held at the Knights of Pythias Hall in Madison, February 6 and 7, 1895. The meeting was called to order by the President, Dr. Woodford, at 2 P.M.

Roll-call showed eight members present, Drs. Arpke, Clark, Laws, Leech, Ormond, Roub, E. D. Roberts, and Woodford, and one visitor, Dr. T. W. Watson.

The minutes of the last meeting were read and approved. The annual reports of the Secretary and Treasurer were read and adopted.

On motion the question of patenting the seal was laid over for a year.

Charges having been preferred against Dr. S. G. Binger by Dr. J. F. Roub, it was moved and carried that he be notified by the Secretary to appear before the Executive Board and show cause why he should not be expelled.

The name of Dr. T. W. Watson was presented for membership and was referred to the Board of Censors, who reported favorably on the application. It was moved and seconded that Dr. Watson be elected a member of the Society. Carried.

The Committee on Legislation presented a bill to regulate the practice of veterinary medicine and surgery. After some slight changes the bill was accepted as corrected.

It was moved and seconded that the Secretary be instructed to send out each year a printed and corrected list of the members to each member. Carried.

Adjourned to 7 P.M.

*Evening Session.* The Society was called to order by the President.

On motion, Drs. Scott, Clark, and Laws were appointed to present the claims of the veterinary bill before the Legislature.

None of the essayists being present, the Society proceeded to the report of cases. Arguments followed in regard to the conditions after death from lightning.

Dr. Roub reported a case of cerebro-spinal meningitis which brought out a general discussion.

Moved and seconded that Dr. Clark be allowed the privilege of bringing his class before the Society during the reading of essays. Carried.

The election of officers resulted in the following-named gentlemen being chosen for the ensuing year: *President*, J. P. Laws, Madison; *Vice-President*, J. R. Kelso, Baraboo; *Secretary*, W. G. Clark, Beaver Dam; *Treasurer*, C. H. Ormond, Milwaukee; *Censors*, J. F. Roub, Monroe, T. A. Watson, Chippewa Falls, and G. Ed. Leech, Milwaukee.

On motion a vote of thanks was tendered Dr. Leech for his faithful and efficient service during his term of office.

Moved by Dr. Leech and seconded by Dr. Laws, that the Secretary be allowed his annual dues and railway fare to and from the association meetings for his services. Carried.

On motion the Society adjourned until 10 A.M., February 7th.

SECOND DAY. *Morning Session.*

The Society was called to order by the President, Dr. Laws. Roll-call.

Minutes of the last meeting were read and adopted.

Moved by Dr. Leech and seconded by Dr. E. D. Roberts that the Society hold the semi-annual meeting at Chippewa Falls on the second Wednesday in August, 1895. Carried.

A resolution was introduced by Dr. Leech to control the Society in the matter of the appointment of the State Veterinarian. On motion the resolution was laid on the table until the next annual meeting.

On motion the following resolution was adopted:

*Resolved*, That the Society instruct the Secretary to request the Governor to create a sentiment in favor of the appointment of a veterinarian on the State Board of Health.

On motion the Society adjourned until 2 P.M.

*Afternoon Session.* The Society was called to order by Dr. Laws at 2 P.M.

Moved and seconded that the central part of the seal be adopted as a lapel button. On motion the original motion was amended, that the Secretary be instructed to ascertain the cost of a gold button of that kind and report at the next meeting. The original motion was then carried.

The names of Christmas Evans and Richard Kuoni were proposed for membership and were referred to the Board of Censors. The Censors reported favorably on the applications. It was moved and seconded that the names be accepted by the Society for membership. Carried.

An interesting paper was then read by Dr. D. Roberts on "Ergotism in Cattle," giving the symptoms and lesions appearing in an outbreak in his practice. It was followed by a spirited discussion of the disease.

On motion the essayist was excused.

Moved by Dr. Leech and seconded by Dr. Arpke, that the members on the programme and not reading papers be requested by the Secretary to read their papers at the next meeting. Carried.

It was moved and seconded that hereafter essayists appointed prepare their papers, and if unable to attend, send them to the Secretary before the meeting, and that the subjects of the essays be included in the invitations. Carried.

On motion a vote of thanks was tendered to the essayist.

The Society then adjourned until the second Wednesday in August, 1895, at Chippewa Falls.

W. G. CLARK,
Secretary.

